# Isoenzyme *N*-Acyl-l-Amino Acid Amidohydrolase NA Increases Ochratoxin A Degradation Efficacy of *Stenotrophomonas* sp. CW117 by Enhancing Amidohydrolase ADH3 Stability

**DOI:** 10.1128/spectrum.02205-22

**Published:** 2022-08-04

**Authors:** Nan Chen, Qingru Fei, Han Luo, Zemin Fang, Yazhong Xiao, Zhengjun Du, Yu Zhou

**Affiliations:** a State Key Laboratory of Tea Plant Biology and Utilization, School of Tea and Food Science Technology, Anhui Agricultural University, Heifei, China; b School of Life Sciences, Anhui University, Hefei, China; University of Minnesota

**Keywords:** food safety, mycotoxin pollution, codegradation, detoxification, isoenzyme, ochratoxin A

## Abstract

Ochratoxin A (OTA) is a potent mycotoxin mainly produced by toxicogenic strains of *Aspergillus* spp. and seriously contaminates foods and feedstuffs. OTA detoxification strategies are significant to food safety. A superefficient enzyme ADH3 to OTA hydrolysis was isolated from the difunctional strain *Stenotrophomonas* sp. CW117 in our previous study. Here, we identified a gene *N*-acyl-l-amino acid amidohydrolase NA, which is an isoenzyme of ADH3. However, it is not as efficient a hydrolase as ADH3. The kinetic constant showed that the catalytic efficiency of ADH3 (*K*_cat_*/K_m_* = 30,3938 s^−1^ · mM^−1^) against OTA was 29,113 times higher than that of NA (*K*_cat_*/K_m_* = 10.4 s^−1^ · mM^−1^), indicating that ADH3 was the overwhelming superior detoxifying gene in CW117. Intriguingly, when gene *na* was knocked out from the CW117 genome, degradation activity of the Δ*na* mutant was significantly reduced at the first 6 h, suggesting that the two enzymes might have an interactive effect on OTA transformation. Gene expressions and Western blotting assay showed that the Δ*na* mutant and wild-type CW117 showed similar *adh3* expression levels, but *na* deficiency decreased ADH3 protein level in CW117. Collectively, isoenzyme NA was identified as a factor that improved the stability of ADH3 in CW117 but not as a dominant hydrolase for OTA transformation.

**IMPORTANCE** Ochratoxin A (OTA) is a potent mycotoxin mainly produced by toxicogenic strains of *Aspergillus* spp. and seriously contaminates foods and feedstuffs. Previous OTA detoxification studies mainly focused on characterizations of degradation strains and detoxifying enzymes. Here, we identified a gene *N*-acyl-l-amino acid amidohydrolase NA from strain CW117, which is an isoenzyme of the efficient detoxifying enzyme ADH3. Isoenzyme NA was identified as a factor that improved the stability of ADH3 in CW117 and, thus, enhanced the degradation activity of the strain. This is the first study on an isoenzyme improving the stability of another efficient detoxifying enzyme *in vivo*.

## INTRODUCTION

As is well known, ochratoxins (e.g., OTA) easily contaminate various agroproducts, such as cereals (e.g., wheat, oats, barley, and soybeans), fruits (grapes and citrus), coco, and coffee beans, and consequently contaminate process foods and feedstuffs (1–3). As reported in toxicological studies, OTA shows teratogenic, potential carcinogenic (group IIB carcinogen), and mutagenic effects, and poses a serious threat to human health (3–5). Development of detoxification methods on mycotoxin-contaminated foods and feedstuffs receives great interest in the food and agriculture fields (6–7). Detoxification studies have shown that OTA is mainly transferred by three possible pathways (i.e., lactone ring opening, producing OP-OTA; isocoumarin ring dechlorinating, producing OTB; or peptidic bond cleaving, producing OTα and l-β-phenylalanine) (8–11). Toxicological evaluation showed that OTα is a much less toxic metabolite compared to the parent chemical OTA, followed by OTB and OTA lactone-opened product (OP-OTA) (10–11).

Among the detoxification methods, biodetoxification by microbial strains and bioenzymes has received much attention for serial merits (environmentally friendly, low cost, and high specificity). However, most of the biodetoxification studies focused on degradation strain screening and characterization (12). In addition, some commercial hydrolases, such as carboxypeptidase and peptidase, were characterized for OTA detoxification, but the efficiencies were relatively low for further industrial development (13–14). Recently, several OTA-detoxifying enzymes (or genes) have been identified, and carboxypeptidases are the most studied. Among the OTA-detoxifying enzymes, the amidohydrolase ADH3 from *Stenotrophomonas* sp. CW117 and ochratoxinase (i.e., OTase) from Aspergillus niger were the most efficient and investigated hydrolases, and OTase was the first crystal structure characterized detoxifying enzyme that was highly significant to understanding the detoxifying mechanism (11, 15). Moreover, the activities of amidohydrolase ADH3, OTase, and the *N*-acyl-l-amino acid amidohydrolase (i.e., AfOTase) from Alcaligenes faecalis, were much more efficient than bovine pancreatic carboxypeptidase (CPA) (the first OTA-detoxifying enzyme) and other characterized hydrolases (11, 16). Other recently isolated carboxypeptidases, such as CP from Bacillus amyloliquefaciens, PJ_1540 from *Acinetobacter* sp., and CP4 from *Lysobacter* sp. showed low activity to OTA transformation (9, 17, 18).

Despite OTA-detoxifying strains and enzymes having been widely investigated, only a few studies focused on OTA-degrading genes (or purified enzymes) screening and characterization. Meanwhile, several studies found that the identified detoxifying enzyme showed much lower activity than the host strain from which the enzyme (or gene) was isolated (9, 17–19). The studies on OTA degradation by *Lysobacter* sp. CW239 found that strain CW239 degrades OTA by joint action of multiple detoxifying enzymes, and the identified carboxypeptidase CP4 showed only limited contribution to OTA degradation in the strain; unfortunately, efficient detoxifying enzymes (or genes) remained unknown (9, 20). Other than *Lysobacter* sp. CW239, no further study tried to illustrate potential reasons for the activity disparity between the identified enzyme and host strain, especially of degradation mechanisms in these degradation strains.

## RESULTS

### OTA-detoxifying enzymes ADH3 and NA from CW117.

**FIG 1:**
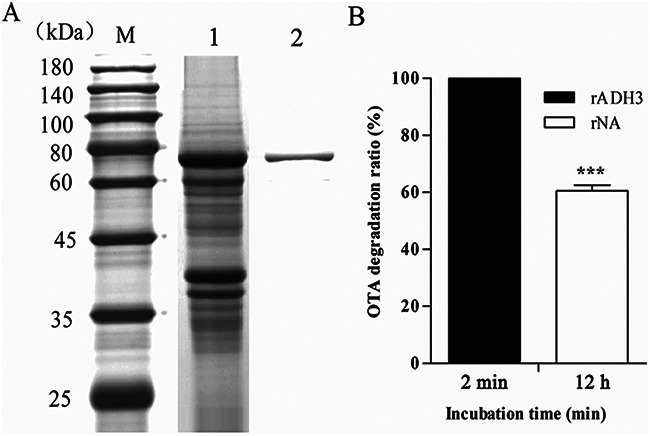
Protein expression and OTA degradation activity. (A) SDS-PAGE analysis of purified rNA (M, marker; lane 1, the unpurified protein sample; lane 2, the purified rNA). (B) Degradation activity of rADH3 and rNA.

As described in a previous publication, 53 hydrolase genes, which may potentially be used for OTA detoxification, were selected for degradation testing from the CW117 genome (GenBank accession no. CP062156.1) (11). Two genes, *adh3* (protein, ADH3) and *na* (protein, NA), were screened and characterized for OTA degradation activity by heterologous protein expression in Escherichia coli BL21 and enzymatic degradation test *in vitro*. Open reading frames (ORFs) of two genes that encode enzymes NA and ADH3 were amplified from the CW117 genome, ligated to vector pGEX-4T-1, and transferred into E. coli BL21 for protein expression. Gene *adh3* has been fully characterized in Luo et al. (11), the repeated ADH3 results were not shown (or only as a parallel comparison) in this study. As shown in Fig. 1, gene *na* (1,317 bp) was cloned from the CW117 genome and recombinant enzyme rNA was expressed by E. coli BL21. Based on the sequence analysis and protein prediction of online programs (Expasy, ProtParam tool), NA is classified as *N*-acyl-l-amino acid amidohydrolase and composed of 438 amino acids, with a predicted molecular weight (MW) of 46.4 kDa and isoelectric point (pI) of 6.1. As shown in Fig. 1A, the purified recombinant *N*-acyl-l-amino acid amidohydrolase (rNA) by GSTrap FF columns showed an apparent MW of 72.4 kDa (including 26 kDa glutathione *S*-transferase [GST]-tag), which was consistent with online predictions. Meanwhile, amidohydrolase ADH3 is composed of 427 amino acids, the theoretical molecular weight is 45.6 kDa, and the isoelectronic point is 6.9. Enzymatic activity tests showed about 60% of OTA was degraded by 0.1 mg/L purified rNA within 12 h, while 25 μg/L OTA was completely degraded by rADH3 in only 120 s with much less protein (1.2 μg/L) (Fig. 1B).

Optimal temperature and pH tests showed that rNA showed greater pH adaptation (pH 2 to 10) than rADH3, but rADH3 showed significantly greater thermal adaptability to rNA (see Fig. S1A and B in the supplemental material). In addition to Li^+^, 0.05 mol/L metal ion Cu^2+^, Zn^2+^, Fe^3+^, Ca^2+^, or Mg^2+^ showed significant inhibition to rADH3 activity; however, metal ion Cu^2+^, Zn^2+^, Fe^3+^, or Ca^2+^ improved rNA activity to OTA degradation, and only Li^+^ and Mg^2+^ showed significant inhibition to rNA activity (Fig. S1C). Two detoxifying enzymes are sensitive to proteases and protein denaturants (1% SDS, proteinase K, or 1% SDS plus proteinase K), but the metal-chelator EDTA showed no inhibition to rADH3 (Fig. S1D). In enzymatic characterization, two detoxifying enzymes NA and ADH3 showed quite different physical and chemical properties from each other, which might enable strain CW117 to degrade OTA in different environmental conditions.

### Phylogenetic results on OTA-detoxifying enzymes and host organisms.

**FIG 2:**
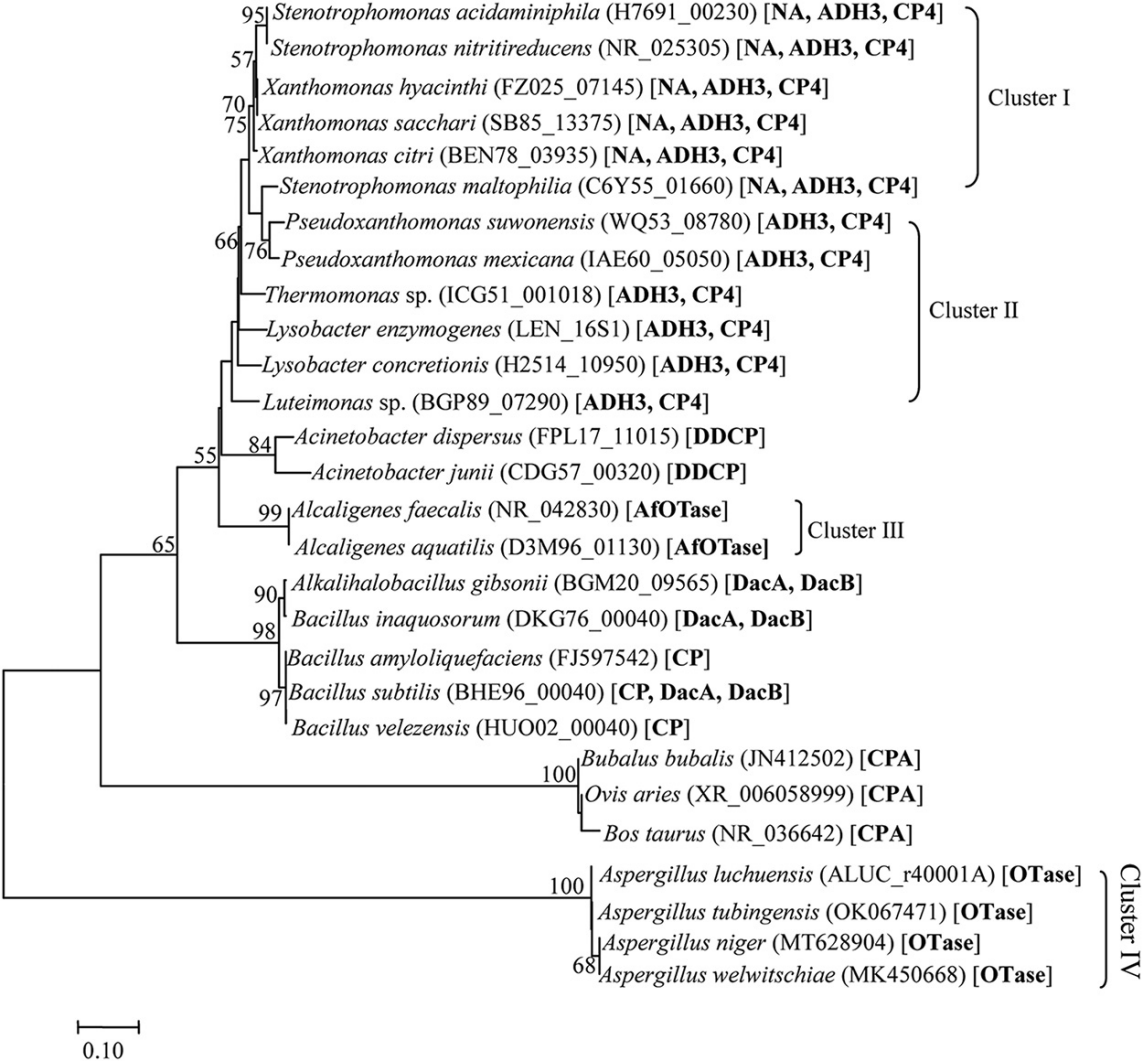
Phylogenetic analysis on the hosts of OTA detoxifying enzymes based on housekeeping genes of each species performed using the neighbor-joining algorithm. Bootstrap values (expressed as percentages of 1,000 replications) of which ≥50% are shown at branching points. Bar, 0.10 substitutions per nucleotide position. The housekeeping genes are 16S rRNA of bacteria, internal transcribed spacer (ITS) of fungi, and 18S rRNA of animals. GenBank accession numbers are shown in parenthesis, and the detoxifying enzymes are shown in square brackets.

Phylogenetic analysis of detoxifying enzymes showed that previously identified OTA-detoxifying enzymes were mainly distributed in two superfamilies of amidohydrolase and carboxypeptidase. Hereinto, detoxifying enzymes with significant activity (*K*_cat_*/K_m_* > 10.0 s^−1^ · mM^−1^) were mainly affiliated with the amidohydrolase superfamily, but detoxifying enzymes with relative low activity were from the carboxypeptidase superfamily (see Fig. S2 in the supplemental material). Detoxifying enzyme NA (gene locus tag H7691_01355) was closest to *N*-acyl-l-amino acid amidohydrolase AfOTase, detoxifying enzyme ADH3 (gene locus tag H7691_12935) was closest to amidohydrolase superfamily OTase, and the two detoxifying enzymes formed separated clades with their closest neighbors (Fig. S2). Phylogenetic analysis of the hosts of detoxifying enzymes found that microbial strains can contain 2 to 3 detoxifying enzymes, but animals contain only one detoxifying CPA and the efficient detoxifying enzymes (e.g., ADH3, OTase, and AfOTase) distributed in four microbial clusters (I to IV) (Fig. 2). Cluster I contains up to three genes, two of which are similar to efficient detoxifying enzymes (NA and ADH3), and cluster II contains two genes, one of which is similar to an efficient detoxifying enzyme (ADH3). Strains of clusters I and II should be the most efficient OTA-detoxifying organisms, which contain more than two genes with at least one gene similar to superefficient enzyme ADH3. The other two clusters (i.e., III and IV) contain only one gene, which is similar to the less efficient enzyme AfOTase or OTase. Interestingly, the organisms from clusters I and II are members of *Xanthomonadales*, and those from cluster III are members of *Burkholderiales*, which are affiliated with phylum proteobacteria (Gram-negative bacteria). In addition to *Aspergillus* spp. of cluster IV, which contain a gene similar to OTase, other efficient OTA degradation organisms almost distribute in phylum proteobacteria. Although detoxifying enzymes from the carboxypeptidase superfamily have been extensively identified and characterized from Gram-positive bacteria (e.g., *Acinetobacter* spp., *Alkalihalobacillus* spp., and *Bacillus* spp.) and some animals (e.g., Bubalus bubalis, Ovis aries, Bos taurus), their activities were substantially limited (Fig. 2).

**FIG 3:**
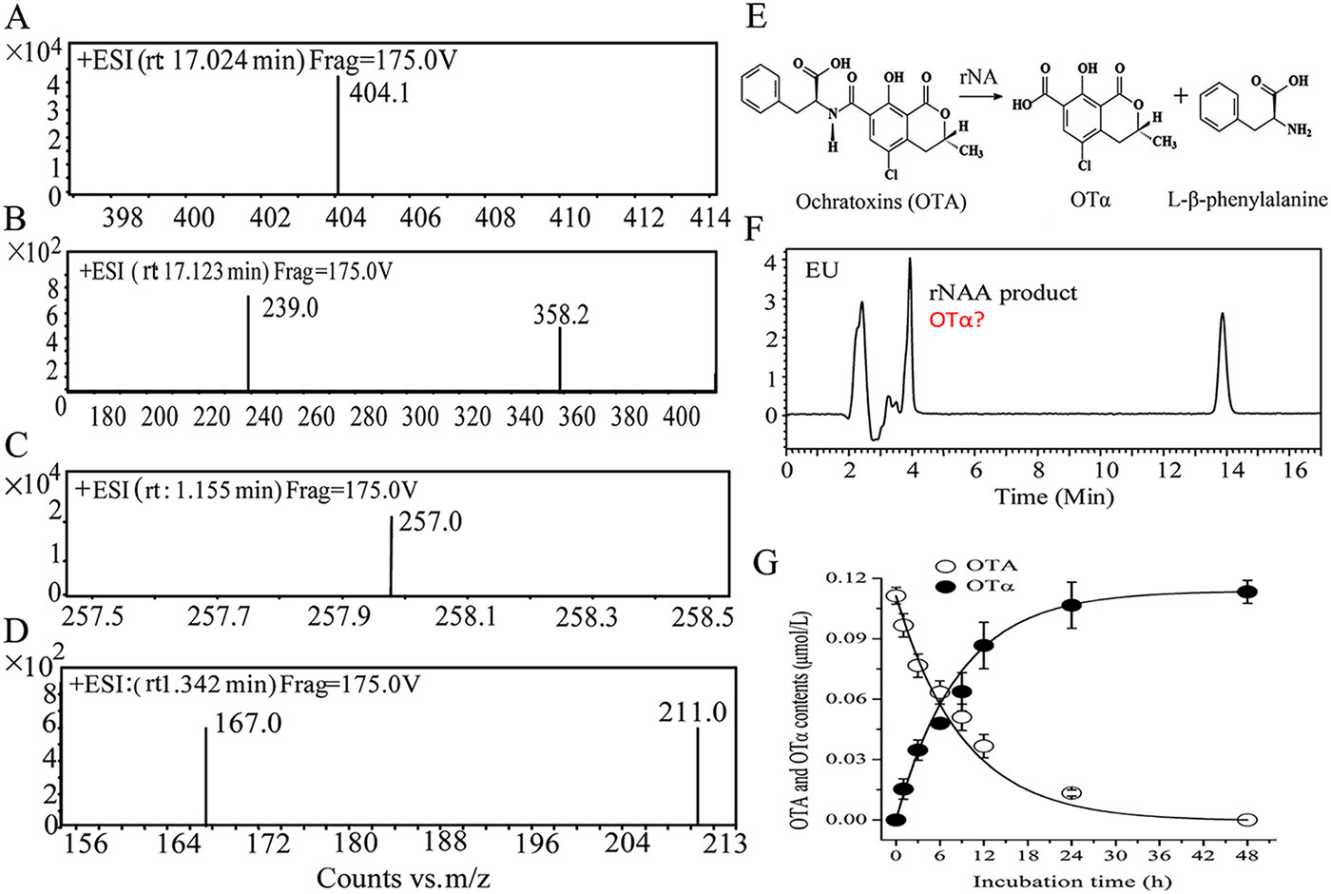
The degradation characteristic of *N*-acyl-l-amino acid amidohydrolase rNA *in vitro* by UPLC-MS/MS under the positive ionization mode. Mass spectra of OTA (A), MS/MS spectra of OTA (B), mass spectra of OTA degradation product (C), MS/MS spectra of OTA degradation product (D), catalyzing mechanism of rNA against OTA (E), HPLC chromatogram of degrade product and OTA residue (F), dynamics of OTA degradation and OTα production (G).

**Degradation dynamics and product identification.** OTA degradation product from rNA was purified using an OchraTest column (Vicam, Milford, MA, USA) and identified by ultraperformance liquid chromatography-tandem mass spectrometry (UPLC-MS/MS) using the same procedures as Luo et al. (11). The OTA degradation product from rNA produced [M + H^+^] at *m/z* 257 as a parent ion from the full scan MS spectrum, and the parent ion produced [M + H^+^] at *m/z* 167 and 211 as daughter ions in MS/MS spectrum, which was consistent with the rADH3-degraded product OTα discussed previously (Fig. 3A to D). Dynamics of OTA degradation and OTα production showed that 0.1 μmol/L OTA was transformed to OTα by 0.1 mg/L rNA within 48 h (Fig. 3E to G), while, rADH3 completed the degradation process within 2 min by 1.2 μg/L protein (11). During OTA degradation, the molar equivalent of OTA residue plus OTα production remained constant (0.1 μmol/L), indicating that the degraded OTA was completely transformed to OTα (Fig. 3G). Kinetic constant showed that *K_m_* values of rNA and rADH3 were 0.0038 mM and 0.000039 mM, and the *K*_cat_*/K_m_* values of the two enzymes were 10.4 and 30,3938 s^−1^ · mM^−1^, respectively. Detoxifying enzyme rADH3 exhibits an extremely high activity to OTA transformation, and the catalytic efficiency (*K*_cat_*/K_m_*) was 29,113 times higher than that of rNA. In addition, the catalytic efficiency of rADH3 was even 210 and 56.7 times higher than those of rOTase from Aspergillus niger and AfOTase from Alcaligenes faecalis, which were previously recognized as the most efficient detoxifying enzymes (11, 15, 16). Other than degradation efficiency, the enzymes NA and ADH3 showed the same degradation mechanism of amide bond hydrolysis (Fig. 3E), and the two hydrolases were the isoenzymes from strain CW117.

**Construction and screening results of mutants and complementary strains.** By PCR amplification, the upstream of gene na (US*_na_*) (792 bp) and downstream of gene na (DS*_na_*) (913 bp) fragments that flank the *na* were amplified, and fragments of US*_na_* and DS*_na_* were ligated by overlap PCR with the US-DS*_na_* fragment of 1,705 bp (see Fig. S3A in the supplemental material). After that, the purified US-DS*_na_* was cloned to pK18*mobsacB* and validated (Fig. S3A). By verifying primers of val1-F/R, the PCR product from the *Δna* mutant was expected to be 1,967 bp, but PCR product from wild-type CW117 was expected to be 3,286 bp (Fig. S3A). As shown in Fig. S3B, the US*_adh3_* (739 bp) and DS*_adh3_* (718 bp) fragments flanking the gene *adh3* were amplified, and the two fragments were ligated by overlap PCR. By verifying primer pair val3-F/R, PCR product from the *Δadh3* mutant (or *Δna-adh3* double mutant) was expected to be 2,292 bp, but PCR product from CW117 (or starting strain *Δna*), which contains *adh3*, was 3,654 bp (Fig. S3B).

Based on mutant construction, the gene (*na* or *adh3*) complementary strain was further constructed and tested for phenotype recovery (i.e., OTA degradation). As illustrated in Fig. S3C and E, the complete ORF of *na* or *adh3* was cloned (1,317 or 1,284 bp), and the recombinant plasmid pSRK-Gm/*na* (or pSRK-Gm/*adh3*) was successfully constructed. The recombinant plasmid pSRK-Gm/*na* or pSRK-Gm/*adh3* was transformed to the *Δna-adh3* double mutant, and the (*Δna-adh3*)/*na* and (*Δna-adh3*)/*adh3* complementary strains were obtained, respectively (Fig. S3D/F).

**FIG 4:**
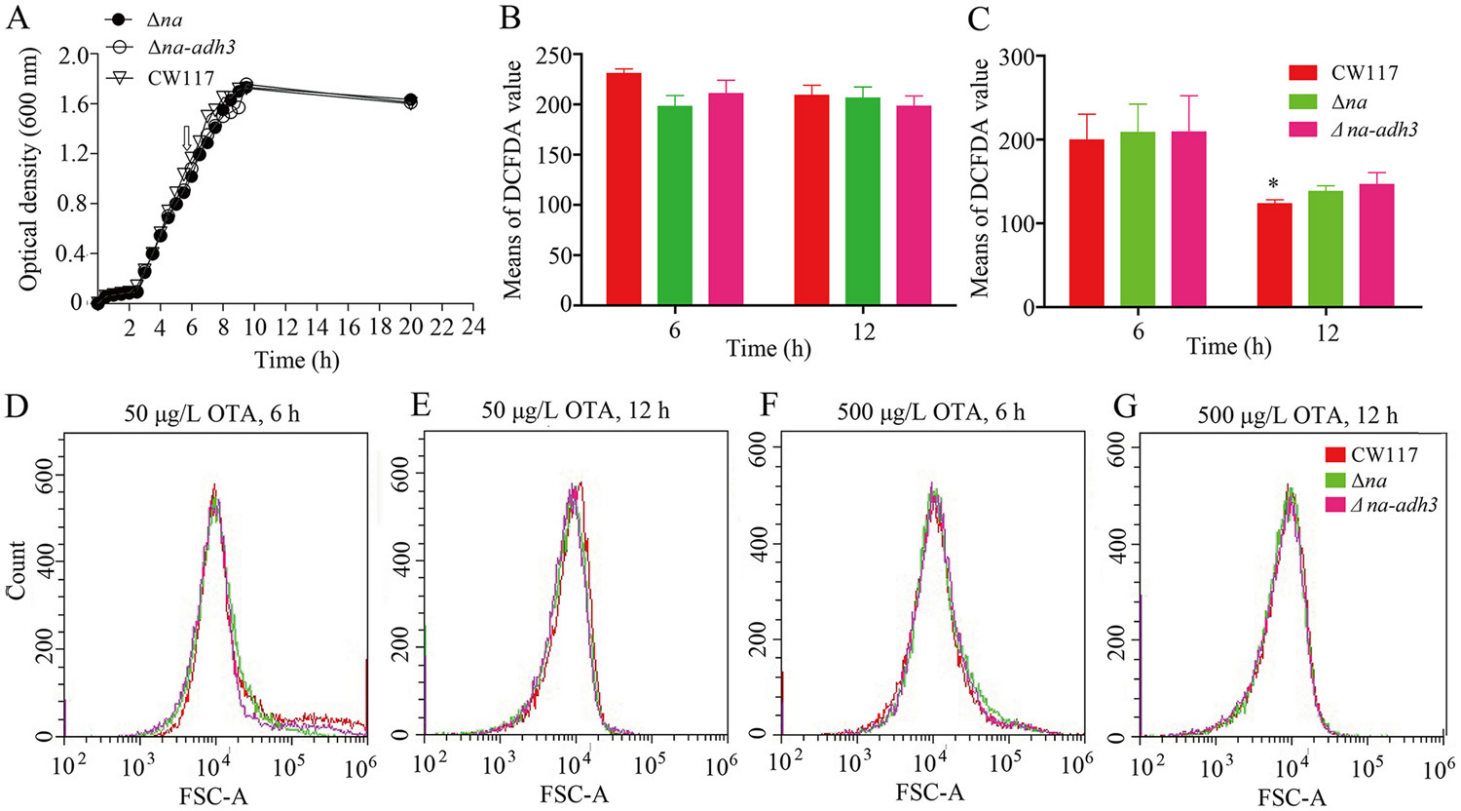
Growth curves and ROS levels of wild-type CW117 and the mutants grown on different conditions. (A) Growth curves and the arrow in growth curves was mid-log phase. (B) DCFDA value of CW117 and mutants incubated with 50 μg/L OTA. (C) DCFDA value of CW117 and mutants incubated with 500 μg/L OTA (Student's *t* test; *, *P* < 0.05, refers to CW117 at the 6th hour versus CW117 at the 12th hour). (D, E, F, G) Fluorescent intensities of CW117, the Δ*na* mutant, and the Δ*na adh3* double mutant cultures, which were stained with H2-DCFDA and analyzed on flow cytometry.

**Growth characters and ROS levels in wild-type and mutants.** As illustrated in Fig. 4A, growth characteristics of the *Δna-adh3* and *Δna* mutants showed no significant difference to wild-type CW117, indicating that genes *na* and *adh3* deficiency did not change the bacterium growth characteristics. Reactive oxygen species (ROS) are recognized as an important physiological indicator for apoptosis and antimicrobial infections, and ROS homeostasis is significant to the health of bacteria (21, 22). Whenever under low or high OTA content, the ROS level of wild-type CW117 showed no significant difference from the *Δna-adh3* and *Δna* mutants (Fig. 4B and G); this result indicated that *na* and *adh3* deficiency did not change ROS homeostasis of CW117. However, at high OTA content, the ROS levels of CW117 and mutants were equally reduced after 12 h of incubation, and a significant difference was observed for strain CW117 (*P* < 0.05), indicating that 500 μg/L OTA showed cytotoxicity to strain CW117.

**FIG 5:**
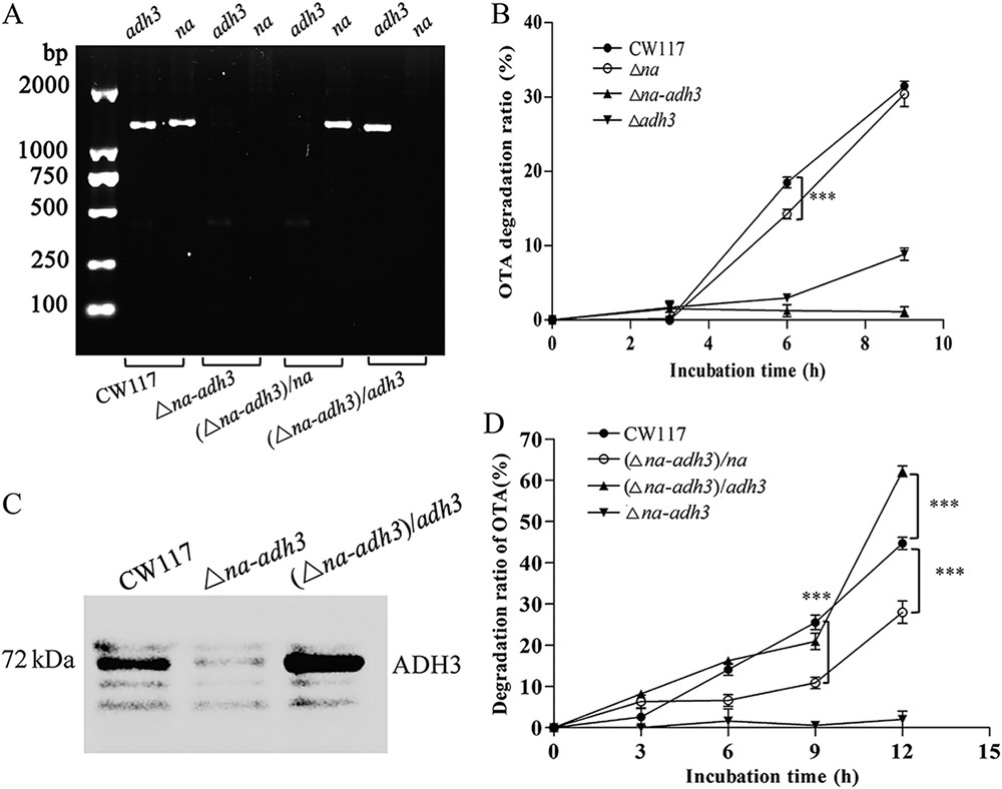
OTA degradation activity on wild-type, mutants, and complementary strains. (A) Mutants and complementary strains validation by PCR (maker, CW117 contains *adh3* and *na*, Δ*na-adh3* mutant without *adh3* and *na*, complementary (Δ*na-adh3*)*/na* strain contains *na* but without *adh3*, and complementary (Δ*na-adh3*)*/adh3* strain contains *adh3* but without *na*). (B) Degradation activity of Δ*adh3*, Δ*na*, and Δ*na-adh3* mutants (Student's *t* test; ***, *P* < 0.001, refers to Δ*na* versus CW117). (C) Δ*na-adh3* mutant and complementary (Δ*na-adh3*)/*adh3* strain validation by Western blotting (10 μg total protein for each sample). (D) Degradation activity of the (Δ*na-adh3*)*/na* and (Δ*na-adh3*)/*adh3* complementary strains [Student's *t* test; ***, *P* < 0.001 refers to the (Δ*na-adh3*)*/na* or (Δ*na-adh3*)/*adh3* complementary strain versus CW117].

**Degradation genes verify *in vivo*.** Before degradation testing, the mutants and complementary strains were further validated by PCR sequencing. As shown in Fig. 5A, the mutants and complementary strains were correctly constructed. The Δ*na* mutant showed similar OTA degradation dynamic to wild-type CW117, except at the time point of the 6th hour, from which the degradation ratio was significantly lower than that of CW117 (*P* < 0.001). Compare to that of the Δ*na* mutant, the degradation activity of the Δ*adh3* mutant was reduced dramatically, and the degradation ratio of the Δ*adh3* mutant was less than 10% at the 9th hour. However, the Δ*na-adh3* double mutant lost OTA degradation activity completely (Fig. 5B). Mutant validation results indicated that genes *na* and *adh3* are the only OTA-detoxifying genes in strain CW117, and *adh3* is much more efficient than *na*. OTA degradation testing of *na* and *adh3 in vivo* was consistent with the activity of detoxifying enzymes rADH3 and rNA *in vitro*.

In complementary strain testing, we found that both the (Δ*na-adh3*)/*na* and (Δ*na-adh3*)/*adh3* strains displayed OTA degradation ability. Thereinto, the (Δ*na-adh3*)/*na* strain, which contains gene *na* recovered only partial activity compared to that of CW117. However, the (Δ*na-adh3*)*/adh3* strain, which contains one gene *adh3* showed a significant higher degradation ratio (*P* < 0.001) than wild-type CW117 at the 12th hour (Fig. 5D). Meanwhile, the expressed ADH3 protein at the 12th hour of the degradation process was examined by Western blotting using the polyclonal antibody prepared previously (see Fig. S4 in the supplemental material). In contrast to OTA degradation results, Western blotting showed that both CW117 and the (Δ*na-adh3*)*/adh3* strain produced detoxifying enzyme ADH3, and the Δ*na-adh3* mutant did not produce ADH3 (Fig. 5C); under 10 μg of total protein loading, the expressed ADH3 protein in the (Δ*na-adh3*)*/adh3* strain that was induced by 0.1 mM isopropyl-β-d-1-thiogalactopyranoside (IPTG) was significantly higher than that of wild-type CW117 (Fig. 5C). Hence, the high expression level of ADH3 should contribute to higher degradation activity for the complementary (Δ*na-adh3*)*/adh3* strain. These results demonstrated that both NA and ADH3 showed OTA degradation activity *in vivo*, and the ADH3 was the crucial detoxifying enzyme that showed much higher activity than NA in strain CW117.

**FIG 6:**
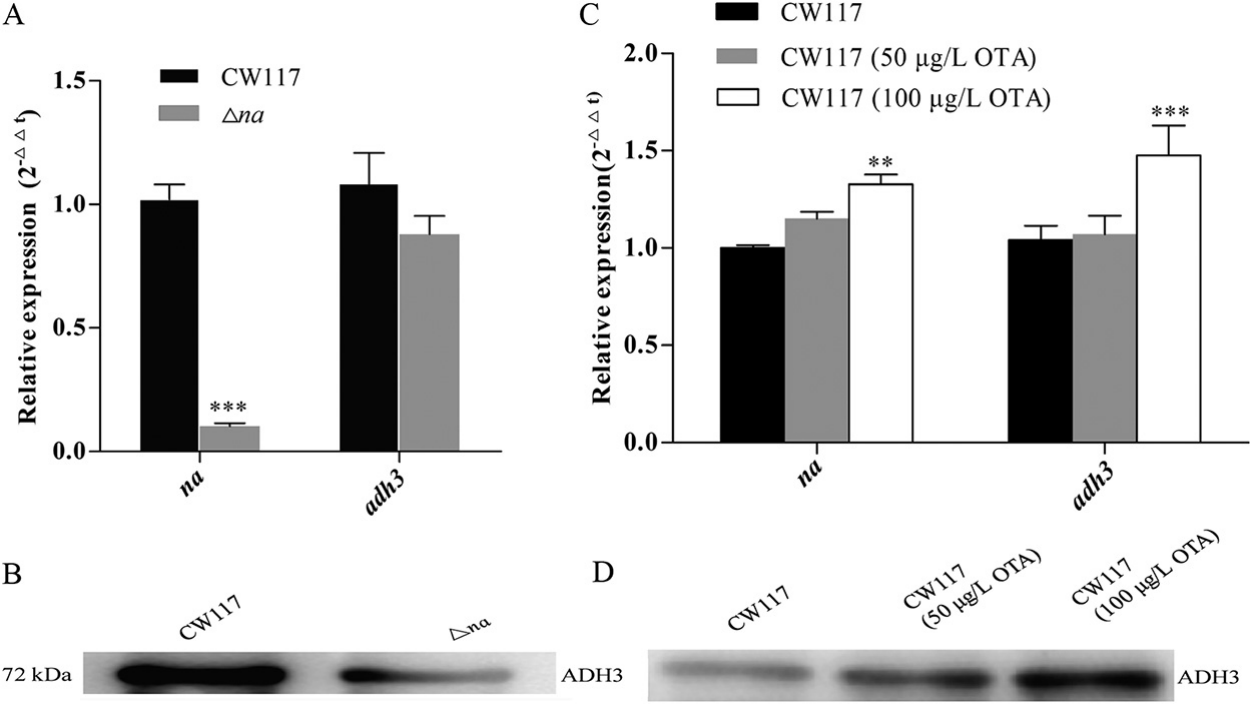
The *na* regulation effects on the expression of gene *adh3*. (A) *na* and *adh3* gene expressions in CW117 and Δ*na* mutant determined by RT-qPCR at the 6th hour during OTA degradation (Student's *t* test; ***, *P* < 0.001, refers to Δ*na* mutant versus CW117; *n* = 12). (B) ADH3 expressed proteins in CW117 and Δ*na* mutant determined by Western blotting at the 6th hour during the OTA degradation (10 μg total protein for each loading). (C) *na* and *adh3* gene expressions by RT-qPCR in OTA-induced CW117 culture (Student's *t* test; **, *P* < 0.01 or ***, *P* < 0.001, refers to 100 μg/L OTA-induced CW117 versus CW117; *n* = 12). (D) ADH3-expressed proteins determined by Western blotting on OTA-induced CW117 culture (10 μg total protein for each sample).

**Joint degradation actions of *na* and *adh3*.** Degradation activity *in vitro* showed that rADH3 catalytic efficiency (*K*_cat_*/K_m_* value) was 29,113 times than that of rNA, and the activity difference of the two enzymes was validated *in vivo*. If we compared the degradation results *in vitro*, the degradation contribution of enzyme NA in strain CW117 should be very limited and can be neglected. However, when compared to wild-type CW117, the degradation activity of the Δ*na* mutant was reduced significantly (*P* < 0.001) in the initial 6 h (*in vivo*) during degradation (Fig. 5B). This result indicated that gene *na* deficiency significantly reduced degradation activity of CW117 in the first 6 h, but the growth character and ROS of the Δ*na* mutant showed no difference from wild-type CW117 (Fig. 4). Corresponding to OTA degradation results, the gene expressions of *na* and *adh3* in the Δ*na* mutant and wild-type CW117 were examined at the 6th hour during the OTA degradation process. Real-time quantitative PCR (qPCR) showed that *na* not expressed in the Δ*na* mutant compared to wild-type CW117; however, the *adh3* expression level in the Δ*na* mutant did not show a significant difference from that of the wild-type CW117 (Fig. 6A). In contrast to RNA levels, the ADH3 protein content in wild-type CW117 determined by Western blotting was significantly higher than that of Δ*na* in the first 6 h. That is, *na* deficiency did not influence the *adh3* RNA level but reduced the ADH3 protein level (Fig. 6B). The comparison result of gene expressions and ADH3 protein contents in the Δ*na* mutant and wild-type CW117 indicated that isoenzyme NA did not influence *adh3* expression but improved the stability of ADH3 in CW117 and thus increased OTA degradation activity of the strain *in vivo*.

In addition, substrate induction characters were examined for the expression of the two genes. When strain CW117 was cultured at the mid-log phase (as shown in Fig. 4A) and induced by 50 and 100 μg/L OTAs for 40 min, the expressions of the two genes (*na* and *adh3*) were upregulated, and significant differences were observed for the 100-μg/L OTA group (Fig. 6C). Compared to RNA levels, the expressed ADH3 protein content of CW117 was gradually enhanced by increasing OTA (Fig. 6D). Substrate induction results by reverse-transcription quantitative PCR (RT-qPCR) and Western blotting indicated that the two genes are substrate inducible, especially under the higher content of 100 μg/L. Moreover, we should realize that *na* and *adh3* are the constitutive expression genes in CW117, and the two genes were constitutively expressed without OTA induction (controls in Fig. 6C and D). That is to say, other than OTA degradation, the two genes might have other physiological functions, such as amino acids metabolism and 2-oxocarboxylic acid metabolism annotated by the Kyoto Encyclopedia of Genes and Genomes (KEGG) database.

## DISCUSSION

In this study, two OTA degradation genes, *na* and *adh3*, were screened and compared from *Stenotrophomonas* sp. CW117, and the degradation activity was verified by *in vitro* and *in vivo* tests. Other than NA and ADH3, no other degradation agent exists in strain CW117 (Fig. 5B). Strain CW117 degrades OTA through amido bond cleavage by the isoenzymes of *N*-acyl-l-amino acid amidohydrolase NA and/or amidohydrolase ADH3, and both of the detoxifying enzymes showed the same degradation mechanism (Fig. 3E), and the pathways that produce product OTα have been recognized as the most effective manner on OTA detoxification, followed by pathways of OTB and OP-OTA as the detoxifying products (8, 10, 11). Degradation verification *in vitro* and *in vivo* from strain CW117 indicated that amidohydrolase ADH3 showed distinguished degradation activity among the identified OTA-detoxify enzymes. As compared to previous results, the *K*_cat_*/K_m_* value of rADH3 (30,3938 s^−1^ · mM^−1^) was about 56 (53,63 s^−1^ · mM^−1^) and 210 (14,44 s^−1^ · mM^−1^) times higher than those of the previously recognized most efficient detoxifying enzymes, rAfOTase and rOTase (11, 15, 16).

Based on an enzymatic activity assay *in vitro*, the *K*_cat_*/K_m_* value of rADH3 was 29,113 times higher than that of detoxifying rNA, indicating the NA contribution to OTA degradation in strain CW117 might be neglected. However, this is not the case for the NA role on OTA degradation *in vivo*. When gene *na* was knocked out from wild-type CW117, the Δ*na* mutant reduced OTA degradation activity in the first 6 h (Fig. 5B). That is, *na* might contribute to OTA degradation in strain CW117 *in vivo* significantly (at least for the first 6 h). In addition, physiological analysis showed that gene *na* knockout did not influence the growth character and ROS level of the strain, and this result indicated that OTA degradation reduction in the Δ*na* mutant could not be ascribed to physiological changes of the CW117. From the RNA expression levels and ADH3 protein contents between wild-type CW117 and the Δ*na* mutant, we found that although *na* knockout did not reduce *adh3* expression level in the Δ*na* mutant at the 6th hour during degradation, the isoenzyme NA can improve the stability of ADH3 in CW117. This result indicated that NA might act as a chaperone that assists the folding of ADH3 or improves ADH3 stability by other unknown mechanisms. When the gene *na* was deleted, the stability of the ADH3 protein reduced, and the ADH3 protein content in the Δ*na* mutant at the 6th hour was lower than that of CW117 (Fig. 6B) and, thus, resulted in OTA degradation reduction.

This study further supported that microbial strains show various mechanisms on OTA detoxification. As discussed above, microbial strains can degrade OTA at least through three possible pathways and produce different degradation products (8–11), and some microbial strains (e.g., lactic acid bacteria and yeast) even detoxify OTA by cell-wall adsorption (23–24). Several studies found that the activity of identified OTA-detoxifying enzyme was much lower than that of the host strain, such as, host strain *Acinetobacter* sp. neg1 and enzyme PJ_1540 (17), strain Bacillus amyloliquefaciens ASAG1 and enzyme rCP (18), strain *Lysobacter* sp. CW239 and enzyme CP4 (9, 20), and Bacillus subtilis CW14 and enzyme DacA (19, 25). The degradation characteristics of the strains shown above might share similar degradation modes as strain CW117, which contains multiple genes for joint degradation, but the exact degradation mechanisms in these degradation strains are rarely focused. Here, two degradation genes were screened from strain CW117, and two genes showed the interactive effect on the pollutant degradation. To the best of our knowledge, this is the first study that clearly illustrated a degradation mechanism on OTA by multiple detoxifying enzymes in a bacterial strain. However, as discussed above, different microbial strains degraded OTA through different pathways and produced various degraded products, indicating different degradation mechanisms. Even though a strain contains multiple degradation enzymes as in this study, the joint degradation mechanism could be different from the synergistic effect seen in strain CW117; other joint mechanisms (e.g., additive effect, antagonism, or potentiation) may exist. Much effort is still needed to investigate the degradation mechanisms on these degradation strains and detoxifying enzymes that have been identified in the past decade. It will be of great significance to pay attention to the investigation of new mechanisms and the screening of more efficient detoxifying enzymes, which are the substantial bases of detoxification development.

## MATERIALS AND METHODS

### Strains, media, and plasmids.

*Stenotrophomonas* sp. CW117 was isolated and preserved in our laboratory (26), Δ*na* and Δ*na-adh3* mutants and (Δ*na-adh3*)/*na* and (Δ*na-adh3*)/*adh3* complementary strains were constructed in this study. Escherichia coli Trans1-T1 and Escherichia coli BL21 were purchased from TransGen Biotech (Beijing, China). Strain CW117, gene mutants, and complementary strains were cultured in nutrient agar (or nutrient broth), and the E. coli strains were cultured in Luria-Bertani (LB) (Difco, KS, USA) as described previously (9). OTA standard (98.0% purity) was obtained from Sigma-Aldrich (St. Louis, MO, USA), and OTα standard (99.0% purity) was obtained from Romer Labs (Washington, MO, USA). Other strains, plasmids, and the PCR primers used in this study are shown in Table 1.

**TABLE 1:** The strains, plasmids, and primers used in this study

Strain, plasmid, or primer	Characterization or oligonucleotide sequence^*a*^^,^^*b*^	Reference/source or endonuclease/primer description
Strain		
CW117	Wild-type degradation strain	This study
pGEX/*na*	*na* expression strain	This study
pGEX/*adh3*	*adh3* expression strain	This study
*Δna*	Gene *na* mutant of CW117	This study
*Δna-adh3*	Double gene (*na* and *adh3*) mutant of CW117	This study
(*Δna-adh3*)*/na*	Gene *na* complementary for *Δna-adh3* (GmR)	This study
(*na-adh3*)*/adh3*	Gene *adh3* complementary for *Δna-adh3* (GmR)	This study
Escherichia coli Trans1-T1 phage resistant	Gene receptive host	TransGen Biotech, Beijing, China
Escherichia coli BL21	Gene expression host	TransGen Biotech, Beijing, China
Plasmid		
pK18*mobsacB*	Allelic exchange vector (KmR)	31, 32
pSRK-Gm	Gene complementary vector (GmR)	34
pGEX-4T-1	Gene expression vector (AmpR)	Geneland, Shanghai, China
pMD18-T	Gene sequencing vector (AmpR)	TransGen Biotech, Beijing, China
Primer		
*na*-F	CGCGGATCCATGATCCGCAAGACCGTTCTGT	BamHI
*na*-R	CCGCTCGAGTCAGCCGGCGCCGCCGT	XhoI
*adh3*-F	CGCGGATCCATGCCGATCCGCCGCCGC	BamHI
*adh3*-R	CCGCTCGAGTCACTGCTTGTAGATCACCCCG	XhoI
*na*-US-F	TGCTCTAGACGAGACGATGCGGGTGTAGC	XbaI
*na*-US-R	CCAGGCATTCCTCGTCGGC	
*na*-DS-F	tgccgacgaggaatgcctggGCCCCGGCCGGCCTTGC	Lowercase indicates sequence for overlap PCR
*na*-DS-R	CCCAAGCTTGCGAATGGCAACCGCAGTGG	HindIII
val1-F	GTCCAACTGCTGTTCGGG	*na* mutant verify
val1-R	GCCTTTTTGTCATTGCTCAT	
com-*na*-F	GGAATTCCATATGATGATCCGCAAGACCGTTCTGT	NdeI
com-*na*-R	CCCAAGCTTTCAGCCGGCGCCGCCGT	HindIII
val2-F	TCGTCGTCGTTCTTCGGGTGC	*adh3* mutant verify
val2-R	TTCCTCGTCTGGCTGGTCTGC	
*adh3*-US-F	GCTCTAGATCCTGGGCGTAGTCGGTGA	Xba I
*adh3*-US-R	GCAACGTGCAAGCGATTGATA	
*adh3*-DS-F	atcaatcgcttgcacgttgcATCTACAAGCAGTGACGACG	Lowercase indicates sequence for overlap PCR
*adh3*-DS-R	CCCAAGCTTCGCGGCAACCTTCACAATC	HindIII
val3-F	GGCATTTGAGAAGCACACG	*na* complementary verify
val3-R	ATACGCAAACCGCCTCTC	
com-*adh3*-F	GGAATTCCATATG ATGCCGATCCGCCGCCGC	NdeI
com-*adh3*-R	CTAGCTAGC TCACTGCTTGTAGATCACCCCG	NheI
val4-F	GACCCTGCCCTGAACCGAC	*adh3* complementary verify
val4-R	CACGACAGGTTTCCCGACTG	
F*_gapdh_*	CCAACCTGAAGTGGGACG	Reference gene *gapdh* RT-qPCR primer
R*_gapdh_*	TTGACGCCGAACACGAAC	Reference gene *gapdh* RT-qPCR primer
F*_na_*	ACCGCCACCACCGACATC	Gene *na* RT-qPCR primer
R*_na_*	GGAGCCGACGAAGAAGAACAT	Gene *na* RT-qPCR primer
F*_adh3_*	CAAGTCCATCGCCACCAC	Gene *adh3* RT-qPCR primer
R*_adh3_*	ACCGAGTTGACCACGCCT	Gene *adh3* RT-qPCR primer

a Restriction sites are underlined. Lower cases indicate sequence for overlap PCR.

b GmR, gentamicin resistance; KmR, kanamycin resistance; AmpR, ampicillin resistance.

### Gene cloning, protein expression, and enzymatic characterization.

The primer pairs of *adh3*-F/R for gene *adh3* and of *na*-F/R for gene *na* as in Table 1 were used for PCR amplification. Restricted sites of BamHI and XhoI were introduced to the 5′ end of the forward and reverse primers of both genes, respectively. Two genes were amplified using the genomic DNA (gDNA) of CW117, and each gene primer pair with PrimeStar max DNA polymerase (TaKaRa, Dalian, China). PCR conditions for gene *adh3* were denatured for 5 min at 98°C followed by 30 cycles of denaturing for 5 s at 98°C, 10 s of annealing at 60°C, 10 s of extension at 72°C, and a final 5-min extension at 72°C. PCR conditions for gene *na* were similar to those for *adh3*, but the annealing temperature was 58°C, and the PCR products were sequenced by Sangon Biotech (Shanghai, China) using an Applied Biosystems DNA sequencer (ABI PRISM 377).

Genes *na* and *adh3* were cloned and ligated to the pGEX-4T-1 vector and finally transformed into E. coli BL21 according to the procedures of Luo et al. (11). The transformant (containing pGEX/*adh3* or pGEX/*na*) was incubated in AmpR LB broth (containing 0.1 mg/mL ampicillin) at 37°C with 160 rpm agitation. Until the culture optical density at 600 nm (OD_600_) reached 0.6, the expression was induced by 0.2 mM isopropyl-β-d-1-thiogalactopyranoside (IPTG) with an additional 8 h incubation at 16°C. After expression, bacterial cells were collected by centrifugation at 8,000 × *g* for 10 min at 4°C, washed twice with phosphate buffer saline (PBS) (pH 7.2), and disrupted by an ultrasonicator (Qsonica Q700; Qsonica Sonicators, Newtown, USA). The recombinant proteins of rADH3 or rNA were purified by GSTrap FF columns (GE Healthcare, MA, USA) according to manufacturer's instructions. The purity of obtained rADH3 (or rNA) protein was evaluated by SDS-PAGE (Bio-Rad, Hercules, USA), and the protein concentration was determined using the bicinchoninic acid (BCA) method (27).

Unless otherwise noted, the rNA solution used in this study was prepared in PBS (pH 7.2) at a concentration of 1.0 mg/mL protein. The degradation mixture for rNA contained 200 μL purified rNA and 1.8 mL PBS (pH 7.2) diluted OTA standard at a final concentration of 25 μg/L, and the degradation mixture was incubated at 42°C for 6 to 24 h. As a parallel test, 200 μL PBS instead of rNA solution was used as a control. OTA degradation activity of rADH3 was determined as in Luo et al. (11). After degradation, OTA residues were examined by an HPLC apparatus (Waters 2695; Waters, Milford, MA, USA) that was equipped with a fluorescence detector (excitation wavelength [λ_ex_] = 333 nm; emission wavelength [λ_em_] = 460 nm). OTA and the degradation product were separated by a C_18_ column (250 × 4.6 mm, 5 μm; Xbridge, Milford, MA USA). The mobile phase contained acetonitrile/water/acetic acid (48:51:1, vol/vol/vol), the flow rate was 1.0 mL/min, and the injection volume was 5.0 μL. OTA-degraded product was purified and identified by LC-MS/MS following the method previously described (9).

The optimal temperature was evaluated by 200 μL rNA mixed with 1.8 mL PBS (pH 7.2) diluted OTA standard (final concentration, 25 μg/L) and incubated for 12 h at 0, 10, 20, 30, 37, 40, 42, 45, 48, and 50 to 100°C with intervals of 10°C. The temperature test on rADH3 was described in Luo et al. (11). The optimal pH (the buffer for pH 2 to 5 was glycine-HCl, the buffer for pH 6 to 7 was PBS, the buffer for pH 8 to 9 was Tris-HCl, and the buffer for pH 10 to 11 was glycine-NaOH), the effects of metal ions (i.e., Cu^2+^, Zn^2+^, Fe^3+^, Ca^2+^, Li^+^, Mg^2+^), the effects of metal chelators (i.e., EDTA and EGTA), and the effects of protein denaturants (SDS or proteinase K, or SDS plus proteinase K) on the two detoxifying enzymes were performed as previously described (9). In each enzymatic test, 200 μL rNA (i.e., 0.2 mg protein) or 2.4 μL rADH3 (i.e., 1.2 μg protein) was used in a 2-mL mixture; the incubation time for rNA was 12 to 48 h, and the incubation time for rADH3 was 2 min.

In a kinetic constant test, 500 μL rNA (i.e., 0.25 mg protein) was mixed with 500 μL OTA solutions with different concentrations (15 μg/L, 25 μg/L, 35 μg/L, 45 μg/L, 55 μg/L, and 65 μg/L). The degradation product OTα was determined by HPLC after reacting at 42°C for 6 h. The same volume of 1*×* PBS (pH 7.2) instead of rNA solution was used as the control. The kinetic constants of *K_m_*, *K*_cat_, and *V*_max_ were calculated following the methods as previously described by a nonlinear regression of Michaelis-Menten equation (11, 28).

### Phylogenetic analysis on detoxifying enzymes and microbial hosts.

The OTA-detoxifying enzymes of this study and those from other peer-reviewed publications with known gene sequences were retrieved from the GenBank database for phylogenetic analysis (11). After multiple sequence alignment by Clustal X 1.8 (29), a phylogenetic tree on the detoxifying enzymes was constructed using MEGA 7.0 (30). Kimura’s two-parameter model was selected to calculate the corrected evolutionary distance, and the neighbor-joining (NJ) algorithm was used for clustering (31–32). Bootstrap analysis was applied to determine tree topology by 1,000 resamplings (33). The potential hosts (e.g., bacteria, fungi strains and animals) of each detoxifying enzyme were obtained by BLAST searches using the gene sequences of detoxifying enzymes. The housekeeping genes of the hosts were retrieved from the GenBank database, and phylogenetic analysis on the hosts were constructed following the same method as detoxifying enzymes.

### Polyclonal antibody preparation and Western blotting.

Recombinant protein ADH3 produced from E. coli BL21 was purified and used for polyclonal antibody production as discussed previously (34). New Zealand White rabbits are immunized by subcutaneous injection of rADH3 (400 μg), which is emulsified in Freund's complete adjuvant, and are boosted every 3 weeks. Serum was collected 10 to 14 days after the last injection and assayed for antibody activity by the indirect enzyme-linked immunosorbent assay (ELISA) method. The antiserum was collected for further affinity purification using rADH3-conjugated agarose beads. The purified antibody was quantified and verified by Western blotting.

In ADH3 Western blotting, CW117 cells were washed twice with PBS, suspended in lysis buffer (50 mM Tris [pH 7.4], 150 mM NaCl, 0.1% 3-[(3-cholamidopropyl)-dimethylammonio]-1-propanesulfonate [CHAPS], 1 mM EDTA, 1 mM NaF, 1 mM Na_3_VO_4_, and protease inhibitors), and disrupted by sonication on ice as above. Protein concentration was determined by the BCA method (27). Ten micrograms (10 μg) of total protein for each sample was subjected to 12% SDS-PAGE electrophoresis, and the gel was transferred onto polyvinylidene difluoride (PVDF) membranes (Millipore, Billerica, MA, USA). The PVDF membrane was blocked for 2 h at room temperature with 5% nonfat milk and incubated with anti-ADH3 antibody at dilutions of 1:1,000 for 2 h at room temperature. Hereafter, the PVDF membrane was washed by TBST (Tris-buffered saline with Tween 20) three times and incubated with corresponding horseradish peroxidase (HRP)-conjugated secondary antibody (ProbeGene, Jiangsu, China) and analyzed as previously discussed (35).

### Gene mutant construction.

Recombinant plasmid pK18*mobsacB*_US-DS*_na_* (or pK18*mobsacB*_US-DS*_adh3_*) construction for gene mutation is shown as in Fig. S5A in the supplemental material, and the scheme of mutants screening is shown as in Fig. S5B. The gene knockout procedures followed previously discussed methods (36–37). Specifically, the upstream (US) and downstream (DS) DNA fragments that were adjacent to gene *na* were amplified by PCR using the PrimerSTAR Max DNA polymerase (TaKaRa, Dalian, China). Two primer pairs, *na*-US-F and *na*-US-R as well as *na*-DS-F and *na*-DS-R, were used for *na*-US and *na*-DS amplification, respectively (Table 1). The PCR conditions for *na*-US (or *na*-DS) were as follows: 10 min denaturing at 98°C followed by 30 cycles of 10 s denaturing at 98°C, 5 s annealing at 63°C, 10 s extension at 72°C, and a final 10 min extension at 72°C. The PCR product (*na*-US or *na*-DS) was purified by agarose gel DNA recovery kit (TransGen, Beijing, China) according to the manufacturer's instruction. Overlapping PCR for US-DS*_na_* linkage was performed by primers of *na*-US-F and *na*-DS-R (Table 1) using the PrimerSTAR Max DNA polymerase, and the purified PCR products US*_na_* and DS*_na_* (1 μL of each) were used as DNA templates. Overlapping PCR conditions were as follows: 5 min denaturing at 98°C followed by 30 cycles of 10 s denaturing at 98°C, 5 s annealing at 61°C, 10 s extension at 72°C, and a final 10 min extension at 72°C. PCR product of US-DS*_na_* was purified and digested with XbaI and HindIII at 37°C overnight. The digest solution consisted of 1.0 μL HindIII, 1.0 μL XbaI, 10.0 μL US-DS*_na_* (or 1 μg pK18*mobsacB*), 5 μL 10*×* M buffer (TaKaRa, Dalian, China), and supplemented double-distilled water (ddH_2_O) to 50 μL. Digested US-DS*_na_* was ligated to suicide plasmid pK18*mobsacB*, which had been digested with the same restriction enzymes, XbaI and HindIII. The ligation solution contained 4 μL digested US-DS*_na_*, 2 μL digested pK18*mobsacB*, and 6 μL solution I DNA ligase, and the ligation was incubated at 16°C for 1 h. After that, 5 μL ligated product was transformed to E. coli Trans1-T1 and screened by LB agar with KanR (i.e., 50 μg/mL kanamycin). Positive clones were identified by PCR sequencing, and the correct recombinant plasmid was defined as pK18*mobsacB*_US-DS*_na_*.

The clone containing the recombinant plasmid was enriched by KanR LB broth and extracted by a Plasmid MiniPrep kit (Axygen, CA, USA). Plasmid pK18*mobsacB*_US-DS*_na_* was transformed into wild-type CW117 by the electroporation method as in Sheng et al. (38). Transformed cells were spread on the nutrient agar with KanR. A single colony on KanR screening agar was transferred to 5 mL KanR nutrient broth, incubated at 37°C with an agitation of 160 rpm. Until the OD_600_ reached 0.8, bacterial cells were collected by centrifugation at 8,000 × *g* and washed twice with sterile nutrient broth and resuspended in nutrient broth. Serial nutrient broth-diluted cells were spread on nutrient agar containing 12% sucrose (i.e., “sucrose screening agar”), and the clones grown on sucrose screening agar were rescreened by KanR nutrient agar. The resulting colonies that can grow on sucrose screening agar but are susceptible to kanamycin are considered to be the *Δna* mutant candidate. Mutant candidate was verified by PCR sequencing with PrimerSTAR Max DNA polymerase and by verify primers val1-F and val1-R (Table 1). The PCR conditions were as follows: 5 min denaturing at 98°C followed by 30 cycles of 10 s denaturing at 98°C, 5 s annealing at 59°C, 10 s extension at 72°C, and a final 10 min extension at 72°C.

The *Δadh3* mutant was constructed and screened by the same protocol of *Δna*. For double gene mutant construction and screening, gene *adh3* was further deleted from the Δ*na* mutant genome by the same protocol as gene *na* knockout from wild-type CW117, producing the Δ*na-adh3* mutant. The PCR primers US*_adh3_* (*adh3*-US-F, *adh3*-US-R), DS*_adh3_* (*adh3*-DS-F, *adh3*-DS-R), and *adh3* knockout verify fragment (val2-F and val2-R) are shown in Table 1.

### Gene complementary strain construction.

Gene *na* or *adh3* was cloned to vector pSRKGm to produce gene recombinant plasmid pSRKGm/*na* or pSRKGm/*adh3* according to the method of Khan et al. (39). The complementary plasmid (pSRKGm/*na* or pSRKGm/*adh3*) was transformed into the cells of the Δ*na-adh3* double gene mutant by electroporation method as previously described to obtain the complementary strain (Δ*na-adh3*)*/na* or (Δ*na-adh3*)*/adh3*. As shown in Fig. S6 in the supplemental material, the *na* open reading frame (ORF) was amplified by PCR using the PrimerSTAR Max DNA polymerase and primers com-*na*-F and com-*na*-R (Table 1). PCR conditions were as follows: 5 min denaturing at 98°C followed by 30 cycles of 10 s denaturing at 98°C, 10 s annealing at 65°C, 20 s extension at 72°C, and a final 5 min extension at 72°C. Purified *na* PCR product and vector pSRK-Gm (gentamicin resistance [GmR]) were double digested by HindIII and NdeI at 37°C overnight, respectively. Digest mixture consisted of 1.0 μL HindIII, 1.0 μL NdeI, 10.0 μL *na* PCR product (or 1 μg pSRK-Gm), 5 μL 10*×* M buffer, and supplemented ddH_2_O to 50 μL. Digested *na* PCR product was ligated to vector pSRK-Gm, which had been digested with the same restriction enzymes. The ligation mixture contained 2 μL digested pSRK-Gm (GmR), 4 μL digested *na*, and 6 μL solution I DNA ligase, and the ligation was incubated at 16°C for 1 h. After that, 5 μL ligated product was transformed to E. coli Trans1-T1 and screened by LB agar with 50 μg/mL gentamicin (GmR). Transformant was enriched by LB broth (GmR), and the plasmid pSRK-Gm/*na* (GmR) was extracted using a plasmid miniprep kit. Recombinant plasmid pSRK-Gm/*na* (GmR) was validated and was transformed into the *Δna-adh3* mutant by electroporation. After that, transformant was spread on GmR nutrient agar. Grown colonies on GmR nutrient agar were the complementary candidate (Δ*na-adh3*)*/na*. The candidate was verified by PCR sequencing with PrimerSTAR Max DNA polymerase, and the primers of val3-F and val3-R. The PCR conditions were the same as the gene *na* clone from the CW117 genome.

The *adh3* complementary strain, (Δ*na-adh3*)*/adh3* strain, construction followed the same procedures as gene *na*. The PCR primers of gene *adh3* were com-*adh3*-F and com-*adh3*-R, and PCR primers for *adh3* complementary strain verification were val4-F and val4-R (Table 1).

### OTA degradation tests on mutants and complementary strains.

Before degradation tests, wild-type CW117, mutants, and complementary strains were further verified by PCR sequencing. Meanwhile, the *adh3* expression levels in wild-type CW117, the Δ*na-adh3* double mutant, and the (Δ*na-adh3*)*/adh3* complementary strain were examined by Western blotting during OTA degradation at the 12th hour.

In mutant degradation tests, strain CW117 and mutants were inoculated into nutrient broth with a final OTA concentration of 50 μg/L. The degradation tests were performed as in Wei et al. (9), and samples were collected at hours 0, 3, 6, and 9 for OTA residue analysis. In complementary strain degradation tests, wild-type CW117 and the Δ*na-adh3* double mutant were used as controls. The control and complementary strains were inoculated into nutrient broth with a final OTA concentration of 50 μg/L. The degradation tests were performed as previously described, but 0.1 mM IPTG was added to the complementary stains to induce complementary gene expression (39). Samples were collected at hours 0, 3, 6, 9, and 12 for OTA residue analysis.

### Growth curves and reactive oxygen species determination.

Growth curves of CW117 and mutants were determined by fresh bacterial culture according to the method of Qian et al. (20). For ROS evaluation, fresh culture of wild-type CW117, the *Δna* mutant, or the Δ*na-adh3* double mutant was inoculated to nutrient broth with different OTA contents (50 and 500 μg/L), and the cultures were collected at 6th and 12th hours for ROS determination. Bacterial cells (1 mL) were collected and further incubated with 1.0 mL (10 μM) 2’,7’-dichlorodihydrofluorescein diacetate, (H2-DCFDA) (BioSharp, Beijing China) for 30 min at 37°C. After incubation, the bacterial cells were washed twice with ultrapure water and resuspended in 1 mL PBS (pH 7.2) for the following fluorescence examination. As a positive control, the bacterial cells pretreated in 1.0 mL Rosup reagent (BioSharp, Beijing China) for 1 h at 37°C were collected by centrifugation and used for the following H2-DCFDA incubation and fluorescence examination. As the negative control, the bacterial cells were washed twice with ultrapure water and resuspended in 1.0 mL PBS (pH 7.2) for H2-DCFDA fluorescence examination. H2-DCFDA fluorescence was examined by flow cytometry (CytoFLEX; Beckman Coulter, CA, USA) following the procedures as previously described (21).

### Degradation gene expressions during OTA degradation.

For joint degradation evaluation, gene expressions (including *na* and *adh3*) were determined and compared at the 6th hour between wild-type CW117 and the Δ*na* mutant during the OTA degradation process. In addition, bacterial strain CW117 under the mid-log phase was induced by 50 and 100 μg/L substrate OTA for 40 min, respectively, and collected for two genes’ OTA induction character examination. RNA was extracted by RNAprep pure bacteria kit (TransGen, Beijing, China), and reverse transcription was performed by FastKing RT kit (TransGen, Beijing, China) according to the manufacturer's instructions. Gene expression levels were examined by reverse-transcription quantitative PCR (RT-qPCR) using SuperReal PreMix Plus (SYBR green) from TransGen Biotech (Beijing, China) following the protocols of Rocha et al. (40). The primer pairs F*_na_*, R*_na_* and F*_adh3_*, R*_adh3_* were used for the amplification of two genes, and the primers of F*_gadph_* and R*_gadph_* were used for gene GAPDH (glyceraldehyde-3-phosphate dehydrogenase) as an internal reference (Table 1). PCR conditions were as follows: 2 min denaturing at 95°C followed by 39 cycles of 5 s denaturing at 95°C, 30 s annealing, and extension at 60°C, and continued for 1 cycle of 5 s denaturing at 95°C, 5 s annealing, and extension at 65°C. Other than RNA examination by RT-qPCR, the expression levels of superefficient enzyme ADH3 in CW117 or the Δ*na* mutant were further determined by Western blotting.

### Statistical analysis.

Unless otherwise noted, the assays in this study were performed in triplicate. Analysis data are shown as the mean ± standard deviation (SD). Student’s *t* test was selected for statistical analysis. Significant difference was accepted at a *P* value of <0.05.
