# Supplementary material for: Isoenzyme N-Acyl-l-Amino Acid Amidohydrolase NA Increases Ochratoxin A Degradation Efficacy of Stenotrophomonas sp. CW117 by Enhancing Amidohydrolase ADH3 Stability
Source: Microbiol Spectr. 2022 Aug 4;10(4):e02205-22. doi: 10.1128/spectrum.02205-22 (PMC9430628; doi:10.1128/spectrum.02205-22)
Supplement: Supplemental file 1 — Supplemental material. Download spectrum.02205-22-s0001.pdf, PDF file, 1.5 MB [file spectrum.02205-22-s0001.pdf]

Supplementary information for

Isoenzyme *N*-acyl-*L*-amino acid amidohydrolase NA increases ochratoxin A degradation efficacy of *Stenotrophomonas* sp. CW117 by enhancing amidohydrolase ADH3 stability

Nan Chen[1¶], Qingru Fei[1¶], Han Luo[1], Zemin Fang[2], Yazhong Xiao[2], Zhengjun Du[1], Yu Zhou[1*]

[1] State Key Laboratory of Tea Plant Biology and Utilization, School of Tea and Food Science Technology, Anhui Agricultural University, Heifei 230036, China

[2] School of Life Sciences, Anhui University, Hefei 230039, China

[¶] These authors contributed equally to this work

*Correspondence Author:

Yu Zhou:

microbes@ahau.edu.cn

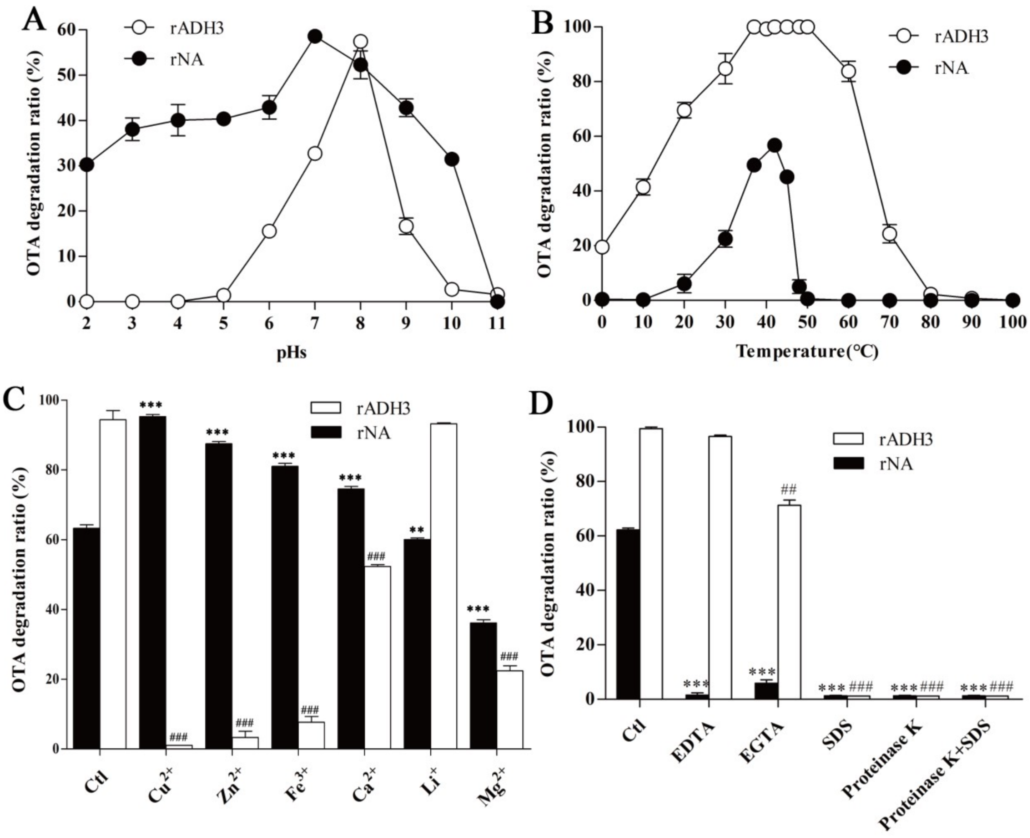

Figure S1. Degradation characteristics of two detoxify enzymes. **A,** pH evaluation and optimal pH; **B,** temperature evaluation and optimal temperature; **C,** metal ions (0.05 mol/L) effect on degradation activity; **D,** metal-chelator and protein denaturant effects on degradation activity.

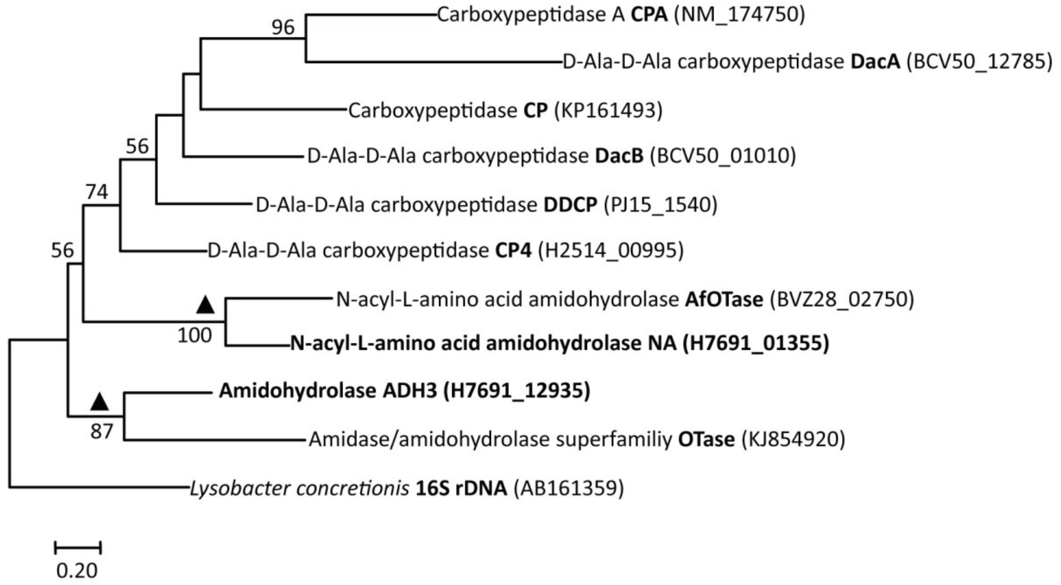

Figure S2. Phylogenetic analysis based on detoxify genes performed using the neighbor-joining algorithm, and showing the relationships of the identified OTA detoxify enzymes. Bootstrap values (expressed as percentages of 1000 replications) ≥50% are shown at branching points. Bar, 0.20 substitutions per nucleotide position. The 16S rRNA gene sequence of of *Lysobacter concretionis* was selected as outgroup. ▲represents the clade of efficient OTA detoxify enzymes.

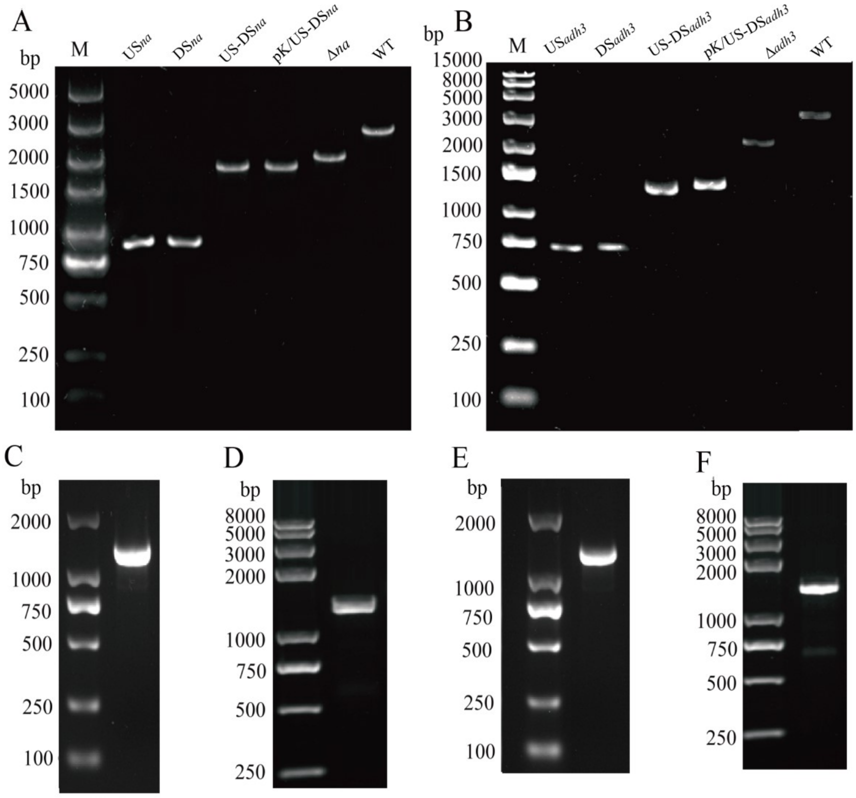

Figure S3. Mutants and gene complementary strains construction for gene *na* and *adh3*. A, *na* mutant construction (M, DNA marker; US$_{na}$, US$_{na}$ fragment; DS$_{na}$, DS$_{na}$ fragment; US-DS$_{na}$, linked US-DS$_{na}$ fragment; pK/US-DS$_{na}$, US-DS$_{na}$ fragment on recombinant plasmid pK18*mobsacB*_US-DS$_{na}$; Δ*na*, *na* mutant; WT, wide-type CW117); **B,** *adh3* mutant construction (M, DNA marker; US$_{adh3}$, US$_{adh3}$ fragment; DS$_{adh3}$, DS$_{adh3}$ fragment; US-DS$_{adh3}$, linked US-DS$_{adh3}$ fragment; pK/US-DS$_{adh3}$, US-DS$_{adh3}$ fragment on recombinant plasmid pK18*mobsacB*_US-DS$_{adh3}$; Δ*adh3*, *adh3* mutant; WT, wide-type CW117); **C,** PCR product of gene *na* from CW117; **D,** PCR product of gene *na* from complementary strain (Δ*na-adh3)/na*; **E,** PCR product of gene *adh3* from CW117; **F,** PCR product of *adh3* from complementary strain (Δ*na-adh3)/adh3*.

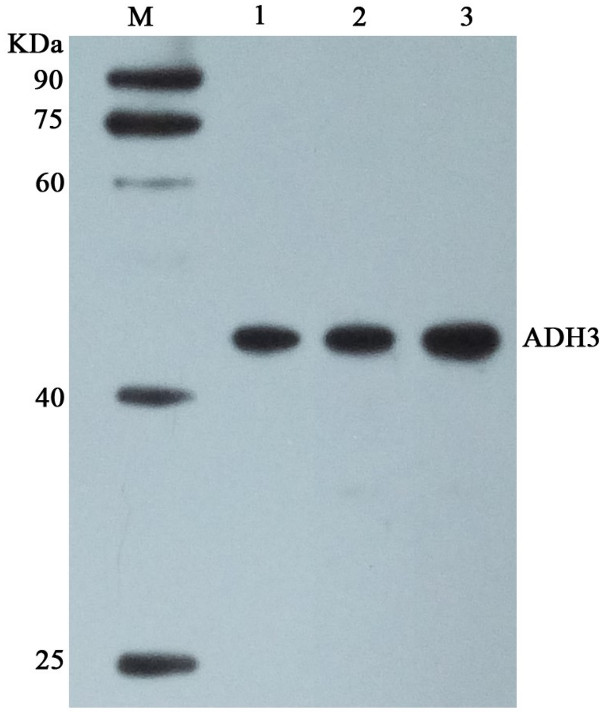

Figure S4. Validation of ADH3 rabbit polyclonal antibody by western blotting. Dilution factor on primary antibody (ADH3 Rabbit Polyclonal Antibody) was 1:1000, on second antibody [Goat Anti-Rabbit IgG (H+L)] was 1:5000 (M, protein marker; lane 1, 1.0 ng rADH3 protein; lane 2, 2.0 ng rADH3 protein; lane 3, 5.0 ng rADH3 protein).

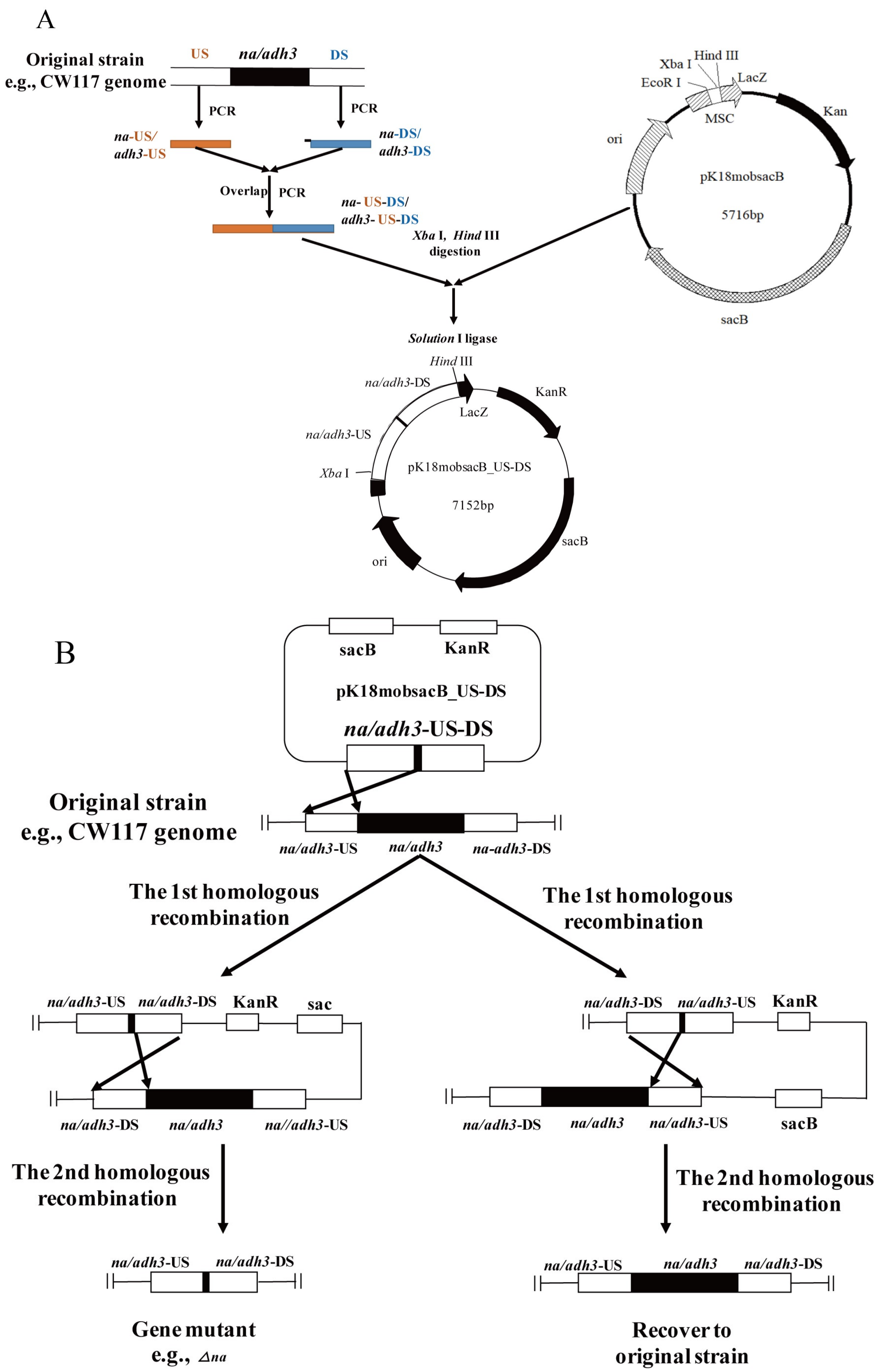

Figure S5. Schematic description of gene mutation by suicide plasmid pK18*mobsacB*. A, recombinant plasmid pK18*mobsacB*_US-DS construction; B, gene knockout and mutant screening.

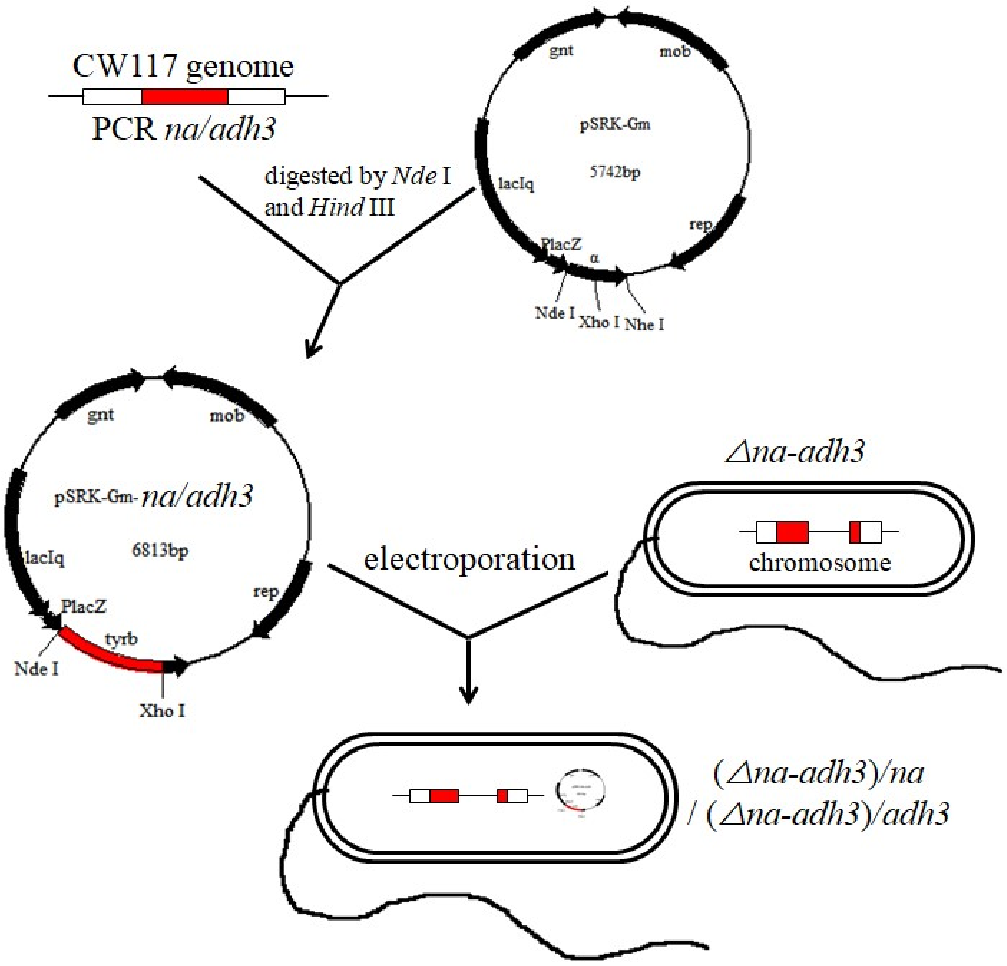

Figure S6. Schematic description of gene complementary strain construction for mutant △*na-adh3*.
